# Myocardial Bridging of Mid-left Anterior Descending Artery (LAD) Presenting As Transient Left Bundle Branch Block During Nuclear Stress Test

**DOI:** 10.7759/cureus.54654

**Published:** 2024-02-21

**Authors:** Kai Shiang Lin, Adam Kurnick, Ridhima Goel, Igal Gorbut, Adam Friedman, Ezra Schrem, Samy I McFarlane, Inna Bukharovich

**Affiliations:** 1 Internal Medicine, State University of New York Downstate Medical Center, Brooklyn, USA; 2 Medicine, State University of New York Downstate Health Sciences University, Brooklyn, USA; 3 Internal Medicine, Saba University School of Medicine, Saba, NLD; 4 Cardiology, State University of New York Downstate Medical Center, Brooklyn, USA; 5 Cardiology, Kings County Hospital Center, Brooklyn, USA

**Keywords:** regadenoson, myocardial bridge, left bundle branch block, nuclear stress test, acute coronary syndrome

## Abstract

Transient left bundle branch block occurring during a nuclear stress test in the setting of myocardial bridging is a relatively rare finding. We report a case of a 75-year-old male who presented with typical stable angina. Serial troponins were negative, and the electrocardiogram revealed normal sinus rhythm with left ventricular hypertrophy and T-wave inversions in the lateral leads. The nuclear stress test was non-ischemic but showed a transient left bundle branch block associated with chest pain and shortness of breath that occurred right after the administration of regadenoson. Coronary angiography revealed non-obstructive coronary artery disease and a mid-LAD myocardial bridge.

## Introduction

The incidence of left bundle branch block (LBBB) during stress testing is relatively rare and occurs in fewer than 1% of patients [1]. An electrocardiographic (ECG) abnormality characterized by a defect in the conduction of electrical impulses down both the anterior and posterior left fascicles of the His-Purkinje system, LBBB can be seen in both healthy patients and in patients with heart disease. One such heart disease is myocardial bridge (MB), a common and generally benign congenital anomaly in which a portion of an epicardial coronary artery is tunneled through the myocardium under an overlying muscular bridge. An isolated LBBB is usually of little concern but can be more consequential when associated with chest pain, syncope, and heart failure [1].

Cardiac stress testing, a non-invasive method of assessing coronary perfusion, cardiac function, myocardium viability, and exercise capacity, is commonly used to evaluate patients with symptoms suggestive of chronic coronary syndrome [2]. One method of stress testing is with radionuclide myocardial perfusion imaging (rMPI), which involves the administration of an intravenous radioactive tracer with subsequent capturing of gamma photons via single-photon emission computer tomography (SPECT) or positron emission tomography (PET). Myocardial perfusion images are obtained at rest and after stress induced by exercise or pharmacological agents, and both images are then compared to detect myocardial perfusion, viability, and global left ventricular systolic function, all of which help signify the presence or lack thereof as well as the extent of coronary artery disease (CAD) [3]. The method of stress chosen for our patient is pharmacologic via regadenoson, a commonly used vasodilatory agent that operates by way of adenosine receptors and induces stress largely via the phenomenon known as coronary steal.

In this report, we present a rare case of regadenoson-induced LBBB in the setting of myocardial bridging in a patient undergoing nuclear stress testing. We highlight the clinical presentation, scintigraphy findings, putative mechanisms, and the management and prognostic importance of transient LBBB in this clinical scenario.

## Case presentation

**Figure 1:**
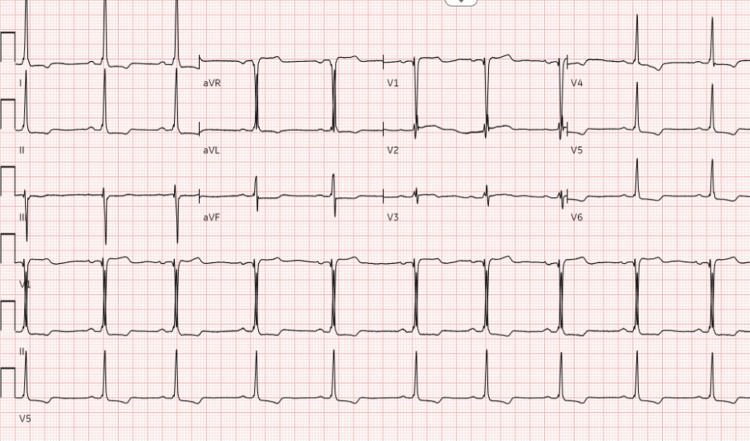
ECG on admission. Normal sinus rhythm, left ventricular hypertrophy, T-wave inversions in leads I, II, V4-V6.

A 75-year-old man presented to the emergency department with four days of intermittent severe chest pain radiating to his left neck and shoulder that was exacerbated by exercise and improved with rest. He reported these pains being similar to prior episodes, wherein he had coronary angiography at a different hospital. He also reported significant dyspnea after walking 10-15 steps, leg swelling, and burning epigastric discomfort while eating, although he denies orthopnea, paroxysmal nocturnal dyspnea, wheezing, palpitations, nausea, vomiting, or lightheadedness. On arrival, his vital signs were notable for a blood pressure of 142/72 mmHg, heart rate of 91 per minute, temperature of 99.3 ºF, respiratory rate of 19, and oxygen saturation at 98% on room air. Laboratory workup revealed troponin of 0.010 ng/mL (normal range: ≤0.010 ng/mL), pro-B-type natriuretic peptide of 97 pg/mL (normal range: ≤ 450 pg/mL), glucose of 150 mg/dL, and hemoglobin of 13.1 g/dL. Electrocardiogram (ECG) revealed normal sinus rhythm, left ventricular hypertrophy, and T-wave inversions in leads I, II, V4-V6 (Figure 1). 

Past medical history

CAD, hypertension, hyperlipidemia, non-insulin-dependent diabetes mellitus type 2, and benign prostatic hyperplasia. Home medications include amlodipine, aspirin, atorvastatin, enalapril, metoprolol succinate, and metformin.

Investigations

**Figure 2:**
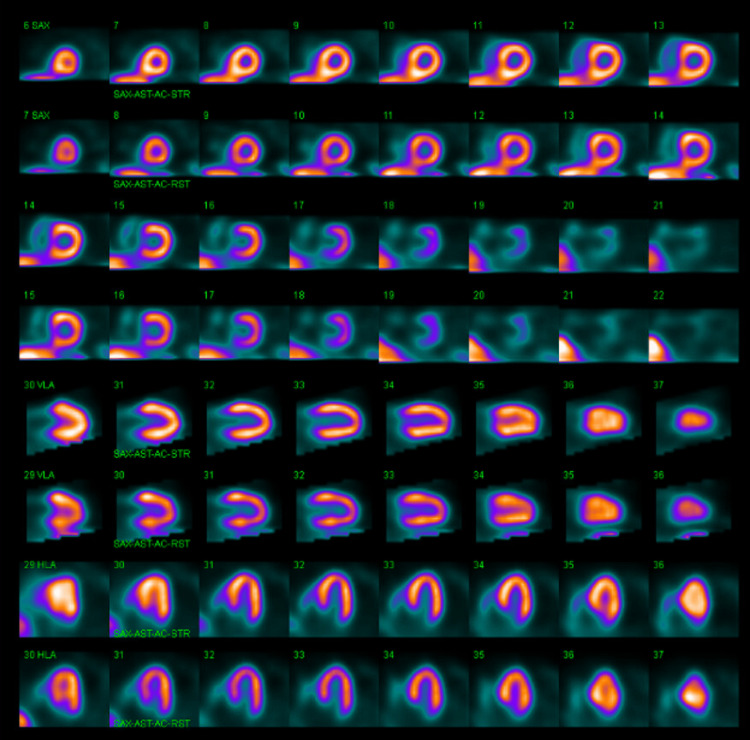
Nuclear stress test. No ischemic perfusion defects, areas of infarction, or regional wall motion abnormalities.

**Figure 3:**
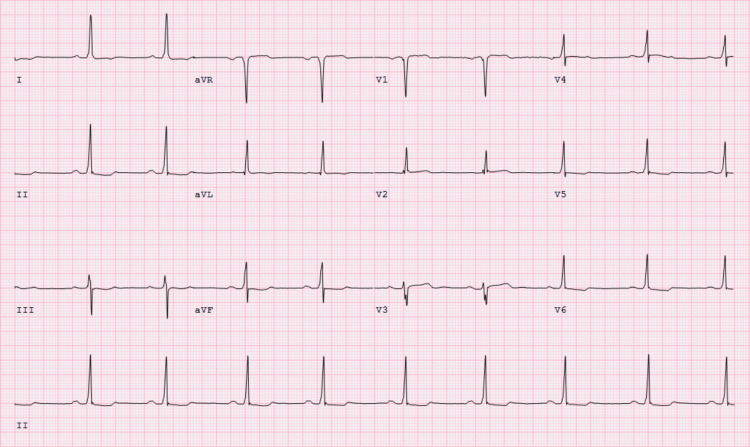
Serial ECGs, 1 of 3, before regadenoson administration. Sinus bradycardia with repolarization abnormalities.

**Figure 4:**
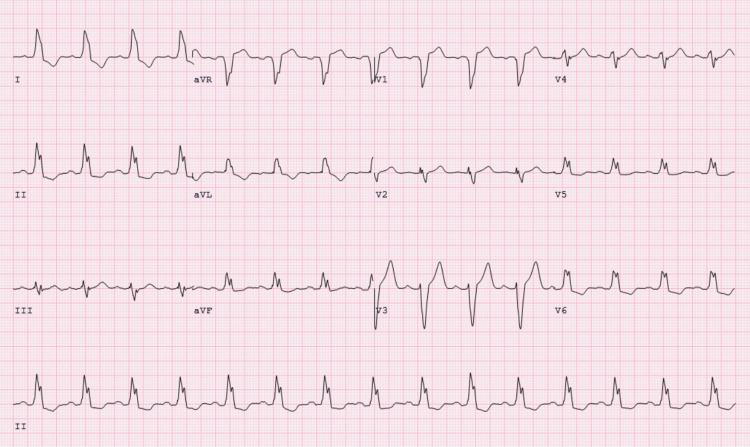
Serial ECGs, 2 of 3, after regadenoson administration. LBBB present. LBBB - left bundle branch block

**Figure 5:**
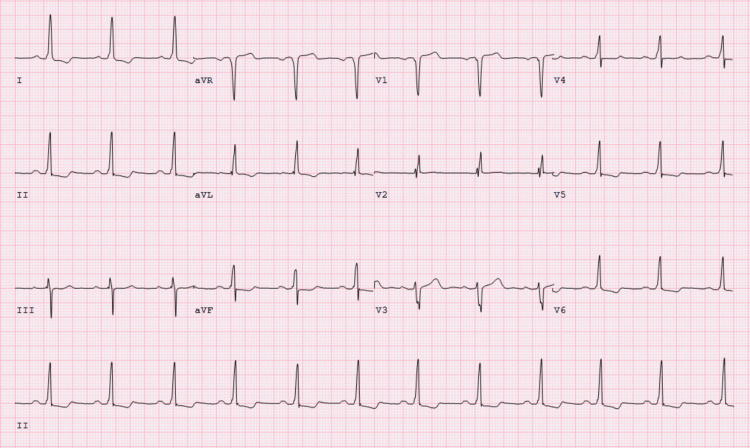
Serial ECGs, 3 of 3, after aminophylline administration. Evident resolution of the LBBB. LBBB - left bundle branch block

**Figure 6:**
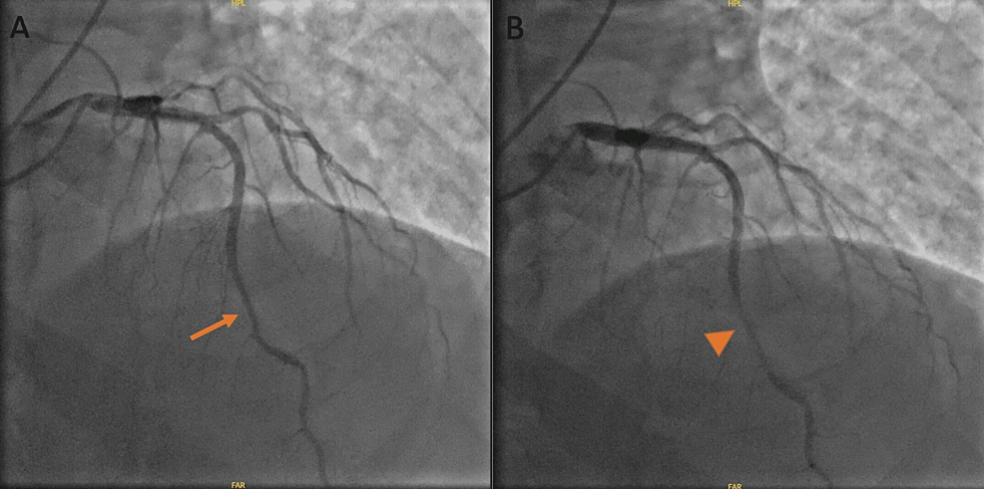
Coronary angiogram demonstrating mild, non-obstructive CAD as well as mid-LAD myocardial bridging. (A) LAD in diastole with an arrow pointing to the area of the myocardial bridge. (B) LAD in systole with an arrowhead pointing to the myocardial bridge. LAD - left anterior descending artery; CAD - coronary artery disease

Upon admission, serial troponins were negative, and a subsequent ECG was unchanged from the admission ECG. Transthoracic echocardiogram revealed normal size and function of left ventricle (ejection fraction of 65%) but moderate left ventricular hypertrophy and severely calcified aortic leaflet valves with moderate aortic valve stenosis (aortic valve max velocity (AVmax) 2.92 m/s, max aortic valve (AV) gradient 35 mmHg, aortic valve area (AVA) 1.3 cm^2^) with no regurgitation. No wall motion abnormalities were observed. A nuclear stress test (NST) revealed no ischemic perfusion defects, areas of infarction, or regional wall motion abnormalities (Figure 2). However, during the test, the patient developed a transient LBBB that was accompanied by chest discomfort, dizziness, and shortness of breath immediately after the administration of regadenoson, all of which resolved after the administration of aminophylline (Figures 3-5). Given the patient’s medical history, the transient LBBB and associated symptoms may have occurred in the context of ischemia despite no such findings on NST. Therefore, the patient underwent coronary angiography, which showed mild, non-obstructive CAD and a mid-left anterior descending artery (LAD) MB, with severe calcification of the LAD and mid-LAD MB (Figure 6). The ramus was normal and tortuous, the left circumflex artery was normal, and the right coronary artery had moderate calcification with 20% stenosis.

Management

The patient presented with anginal-type chest pain and was admitted for rule-out of acute coronary syndrome. His chest pain improved with the administration of nitroglycerin 0.3 mg, acetaminophen 650 mg, and ibuprofen 400 mg, and the patient was started on aspirin 81 mg daily, metoprolol succinate 25 mg daily, rosuvastatin 40 mg daily, and losartan 50 mg daily. After the patient’s NST, the patient was initially begun on clopidogrel 75 mg daily, which was later discontinued after the coronary angiogram showed non-obstructive CAD. The patient was ultimately discharged on medical therapy for stable CAD.

## Discussion

MB, a congenital anomaly that occurs when a portion of an epicardial coronary artery is tunneled through the myocardium under an overlying muscular bridge, was first discovered in 1737 on an autopsy performed by Dr. Henric Reyman and was considered a benign cardiovascular anomaly at that time [4]. Although MB is generally asymptomatic in mild cases, it can also be associated with shortness of breath, angina, and arrhythmias. The current prevalence of MB is about 33%-42% and it most commonly affects the mid-LAD with approximately 67%-98% of cases involving the LAD [1,5,6]. Integral to its pathophysiology is the compression of the bridged artery during systole, which results in a delay in luminal expansion during early diastole, leading to arterial spasm. Presenting symptoms can mimic those of myocardial ischemia, although the underlying etiology is primarily the transient interruption in regular coronary flow rather than underlying atherosclerosis. This transitory nature of the ischemia is thought to irritate the endocardium and is especially exaggerated in states of increased sympathetic tone [7].

Anatomically, MB can be categorized as “superficial” or “deep.” The superficial variant involves the artery crossing the muscle perpendicularly or at an angle, and the deep variant by the artery deviating towards the interventricular septum and muscle, arising from the right ventricle and crossing transversely, oblique, or helically. It is thought that compression of the “deep” variant is what commonly leads to symptomatic ischemia as described above [8]. Aside from causing symptoms, MB is also associated with nonspecific ECG changes, including ST-segment or QTc anomalies, that can occur concomitantly with symptomatic chest pain. These ECG changes are thought to result from transient ischemia secondary to coronary compression by the myocardium during systole [9].

Despite being a distinct etiology for ischemic chest pain, MB itself can also be a risk factor for accelerated atherosclerosis, which tends to occur most commonly in the proximal coronary segment while sparing the bridged section. One theory for this unusual phenomenon is that while transitory changes in coronary flow contribute to an increased rate of atherogenesis, the bridged section is likely spared due to shearing forces during compression that leads to the release of vasoactive agents from endothelial and smooth muscle cells [8]. Another theory is that disruption of the normally occurring systolic wave from retrograde flow during systolic compression leads to the occurrence of lower shear stress immediately prior to the MB, resulting in a buildup of atherosclerosis. In addition to causing accelerated atherosclerosis, systolic compression of the bridged artery also leads to the release of pro-inflammatory mediators, and their complex interaction with shearing forces during coronary compression in conjunction with aging and other comorbidities can result in ischemia, left ventricular diastolic dysfunction and hypertrophy.

Testing for MB is most commonly done through CA, which can demonstrate systolic narrowing or “milking” of the vessel associated with “step-down” and “step-up” in the impacted area that demarcates the affected coronary segment (1). This was the method we used to make the ultimate diagnosis of MB, and CA showed mild, non-obstructive CAD and mid-LAD MB.

In addition to MB, we also observed the development of a transient LBBB after the administration of regadenoson during our patient’s NST. Commonly known to be a risk factor for adverse cardiovascular outcomes, LBBB generally develops from alterations in electrical flow secondary to altered mechanics, perfusion, and workload in the setting of cardiac remodeling. Causes include hypertension, acute coronary syndrome, previous myocardial infarction, cardiomyopathy, aortic and mitral valvular disease, cardiac procedures, and interventions. The Framingham Study in 1979 examined 55 patients who developed LBBB over an 18-year period of observation and noted the risk factors for LBBB to be CAD and congestive heart failure; the development of LBBB also appeared to be an independent risk factor for increased cardiovascular mortality in the male gender [10,11]. Another study, published in The American Journal of Cardiology in 1996, screened 110,000 Irish subjects for cardiovascular disease and found 112 of those subjects to have LBBB. After a mean follow-up of 9.5 years, patients with LBBB were found to have a significantly higher risk of developing overt cardiovascular disease and increased cardiac mortality compared to those who did not [12]. Because of its association with adverse cardiovascular outcomes, the development or presence of LBBB requires investigation and opens up a large differential diagnosis that includes MB, an etiology difficult to assess non-invasively. One key reason that makes MB challenging to assess without invasive measures is its similarity in clinical presentation to acute or chronic coronary syndrome. This was certainly the case for our patient, as the distinction was difficult with NST alone, and a subsequent cardiac catheterization was required to rule out coronary ischemia and confirm the presence of MB.

Regadenoson, a selective A2a adenosine receptor agonist first approved by the FDA in April 2008, was used during our patient’s NST to reveal underlying myocardial ischemia through vasodilation of the coronary arteries [13]. A2a receptors are stimulatory guanine nucleotide-binding proteins (G proteins) which, when bound to regadenoson, activate adenylyl cyclase, thereby increasing cyclic adenosine 5T monophosphate (cAMP). This leads to the phosphorylation of protein kinase A (PKA) and the production of membrane hyperpolarization, which results in hyperemia and increased coronary flow [14, 15]. It is the selective agonism for A2a receptors that distinguishes regadenoson from older agents such as adenosine, as the selective agonism is thought to result in an improved safety profile and a lower risk of causing undesirable side effects such as chest pain, bronchospasm, atrioventricular (AV) block, hypotension, and arrhythmias [16]. And yet, this thought has recently been put into question. Recent data comparing the safety between adenosine and regadenoson in patients undergoing cardiac stress tests suggest that regadenoson administration may actually lead to both a higher incidence of arrhythmias and the need for rescue agents such as aminophylline. This may have been the case for our patient, as he not only experienced LBBB but also developed increased heart rate, both of which are known side effects of regadenoson [17]. In particular, our patient’s heart rate went up to a peak of 100 (Figure 4). Because tachycardia and abrupt changes in heart rate are known risk factors for the development of LBBB (i.e., rate-dependent LBBB), it may be difficult to determine the exact cause of LBBB in our patient; regadenoson stress, increased heart rate, or even a combination of the two could all be potential etiologies [18].

Another consideration is underlying coronary ischemia, which is a known risk factor for LBBB, particularly during regadenoson administration. However, this etiology seems less likely in our patient who had a normal NST and a non-obstructive angiogram. Lastly, the occurrence of LBBB without any transient ischemic dilations on NST is a rare presentation. Besides being an independent predictor of progression to permanent LBBB, it is also an independent predictor of CAD, particularly in elderly patients in whom the LBBB was induced at heart rates of 125 or below [19]. Therefore, it appears reasonable to view transient LBBB on NSTs as a strong indicator for further workup with coronary angiography, irrespective of myocardial scintigraphy findings, as was done with our patient.

Our case report adds to the sparse literature that describes the phenomenon of transient LBBB occurring in patients with MB in the setting of non-obstructive coronary artery disease and highlights the importance of thorough cardiac investigation in patients presenting with chest pain and transient LBBB.

## Conclusions

We report the case of an elderly male who presented to the hospital with four days of typical angina. Upon admission, ACS was ruled out, but NST revealed transient LBBB associated with chest pain, dizziness, and shortness of breath after administration of regadenoson. The symptoms and LBBB were terminated after administration of aminophylline. CA showed mild, non-obstructive CAD and a mid-LAD MB. This case report adds to the growing literature that documents the presence of transient LBBB during NST in a patient without significant CAD but with MB.
